# Small RNA expression and strain specificity in the rat

**DOI:** 10.1186/1471-2164-11-249

**Published:** 2010-04-19

**Authors:** Sam EV Linsen, Elzo de Wit, Ewart de Bruijn, Edwin Cuppen

**Affiliations:** 1 Hubrecht Institute-KNAW & University Medical Center Utrecht, Cancer Genomics Center, Utrecht, The Netherlands

## Abstract

**Background:**

Digital gene expression (DGE) profiling has become an established tool to study RNA expression. Here, we provide an in-depth analysis of small RNA DGE profiles from two different rat strains (BN-Lx and SHR) from six different rat tissues (spleen, liver, brain, testis, heart, kidney). We describe the expression patterns of known and novel micro (mi)RNAs and *piwi*-interacting (pi)RNAs.

**Results:**

We confirmed the expression of 588 known miRNAs (54 in antisense orientation) and identified 56 miRNAs homologous to known human or mouse miRNAs, as well as 45 new rat miRNAs. Furthermore, we confirmed specific A to I editing in brain for *mir-376a/b/c *and identified *mir-377 *as a novel editing target. In accordance with earlier findings, we observed a highly tissue-specific expression pattern for all tissues analyzed. The brain was found to express the highest number of tissue-specific miRNAs, followed by testis. Notably, our experiments also revealed robust strain-specific differential miRNA expression in the liver that is caused by genetic variation between the strains. Finally, we identified two types of germline-specific piRNAs in testis, mapping either to transposons or in strand-specific clusters.

**Conclusions:**

Taken together, the small RNA compendium described here advances the annotation of small RNAs in the rat genome. Strain and tissue-specific expression patterns furthermore provide a strong basis for studying the role of small RNAs in regulatory networks as well as biological process like physiology and neurobiology that are extensively studied in this model system.

## Background

miRNAs are ~22 nt-long, single stranded RNA molecules that mediate post-transcriptional regulation of gene expression by directing the RNA-induced Silencing Complex (RISC) to the 3' untranslated region (UTR) of target mRNAs [1,2]. As a result, translation is inhibited and/or the mRNA degraded [3,4]. The target spectrum of a miRNA is mostly defined by the seed, i.e. the 1^st ^or 2^nd ^7 nt, which hybridizes to the target mRNA [5,6]. miRNAs can both act as developmental switches [7-9] or subtly tune expression, when tight regulation of a gene is required [10,11]. Thousands of mRNAs are expected to be under regulatory control of miRNAs [12,13] and the presence or absence of a single miRNA has been shown to affect, albeit modestly, the level of thousands of proteins [14,15]. Thus, miRNAs form a complex regulatory network affecting the majority of genes.

A second developmentally vital class of small RNAs are the *piwi*-interacting (pi)RNAs [16,17], which play a role in the formation of the germ line. In mammals, these ~27 nt ssRNAs are expressed in the reproductive organs, mainly the testis [18,19], where two types can be distinguished. The pre-pachytene piRNAs, which are repeat- and transposon-derived, likely play a role in guiding DNA methylation to repeats, thereby silencing transposons [20] and preventing genome instability. Conversely, the pachytene piRNAs are mostly derived from a selected set of genomic clusters that show a very strong strand bias. The function of these genomic clusters, however, remains elusive [18-21].

To a certain extent, development and homeostasis of organ systems depend on miRNAs [22,23] and piRNAs [24]. The laboratory rat (*Rattus norvegicus*) is a model organism in which organ-systems physiology has been studied for decades [25]. Recent advances in techniques to genetically modify the rat [26-30] enables detailed analyses of rat physiology at molecular levels. Furthermore, well-established genetic systems, such as congenic, consomic and recombinant inbred lines are versatile tools for studying the effect of genetic variation on quantitative traits such as blood pressure [31] or gene expression [32] (corresponding to quantitative trait loci (QTLs) and expression (e)QTLs, respectively). Comprehensive small RNA inventories and profiles are instrumental in such genetical genomics and systems biological approaches, as they serve as a resource for annotation of the genome. Small RNAs are important players in many regulatory processes and are thus important for understanding disease etiology. The rat small RNA inventory described here will also be important for understanding human disease, since many rat models were selected to reflect clinical symptoms [33].

Conserved expression specificity of miRNAs has been described for a number of organ systems or cell cultures, based on (deep) sequencing approaches [34-36]. Simultaneously, species-specific miRNAs have been identified in closely related species [34,36-38], indicating that miRNAs are evolutionary dynamic. The availability of comprehensive species-specific miRNA profiles of different tissues and organ systems is an important requirement for elucidating the biological roles that miRNAs fulfill. More exhaustive profiling will likely improve existing profiles and increase insight in the basis of quantitative and qualitative variations in miRNA expression. We therefore performed digital gene expression (DGE) profiling of small RNAs from six tissues, i.e. brain, liver, spleen, heart, testis and kidney of the BN-Lx and SHR rat inbred strains, the founder strains of the BXH/HXB recombinant inbred panel [39]. We identified 588 known miRNAs (54 in antisense orientation) and 101 new rat miRNAs, originating from 276 and 61 precursor (pre) miRNA loci, respectively. Thirty-one of these pre-miRNAs were not previously characterized in rat, but were found to be homologous to mouse or human loci; 30 novel candidate pre-miRNA loci do not have an apparent homologue in these species. By generating DGE profiles for liver from three individuals from each strain, we observed strain-specific differential miRNA expression for 4 miRNAs. Finally, we identified thousands of piRNAs in the testis samples. The dataset described here greatly contributes to our understanding of miRNA divergence, variation and expression and may be a valuable resource in evolutionary analyses as well as in the interpretation of regulatory networks and functional genomics experiments in the rat.

## Results and discussion

### miRNA identification

We collected the small RNA fraction and prepared small RNA sequencing libraries from 6 different tissues (i.e. whole brain, liver, spleen, heart, testis and kidney) from two rat inbred strains (polydactyly-luxate syndrome brown Norway (BN-Lx) and spontaneous hypertensive rats (SHR), adult males). The libraries were sequenced on the SOLiD platform version 2 (ABI), generating 115 million small RNA sequence reads. Of all raw reads, 41.9 million could be mapped to the rat genome (see Additional file 1, **Table S1 for individual libraries**). The length distribution of the vast majority of small RNA reads was between 18 and 23 nt, corresponding with the expected size for miRNAs. Exceptions to this observation were testis small RNAs, which are generally longer (Additional file 2, **Figure S1A**). In all tissues except testis, the majority of the reads mapped to known miRNAs [40]; small RNAs from testis were mostly derived from repeats or small RNA classes other than miRNAs most likely corresponding to (pre-)pachytene piRNAs [18-21] (Additional file 2, **Figure S1B and see below)**. We aimed to address global profiling as well as RNA editing and for profiling we only included perfectly mapping reads (6.4 million). Each tissue dataset consisted out of approximately 400,000 miRNA reads.

We identified 688 miRNAs, derived from 276 of the known miRNA loci (miRbase v12 [40]), 31 miRNA loci that were homologous to known miRbase miRNAs from either mouse or human and 30 confident novel pre-miRNA loci (Additional files 3, 4, 5 and 6, **Files S1-4)**. We assume that our global expression profiles allowed us to detect most miRNAs. Only eleven miRBase miRNAs (4%), i.e. rno-mir-7b, 147, 291a, 297, 327, 335, 347, 340, 349, 352 and 671, were not detected. This is likely due to the absence of the miRNA in the investigated tissues, or highly specific expression in a limited number of cells. Eight of the novel miRNAs reside in close proximity (< 100 kb) to known miRNAs in the rat. We obtained reads from both the 5' and 3' arm for 73.4% of the pre-miRNAs. Although the arm with the lowest read frequency is likely to represent the miRNA* sequence, it is also clear that the read frequency of small RNAs is subject to capture bias [41] and read frequencies per arm may be misleading. Therefore, we treated miRNAs derived from either hairpin arm as individual entities for further analysis steps. Seed (nt 1-7 or 2-8) overlaps with known miRBase miRNAs showed that the majority of miRNAs is conserved; 515 are conserved in rodents, whereas comparison to all categorized vertebrates shows that 610 seeds are conserved. Likely, the higher number of seed conservation in all vertebrates is due to the poor miRNA annotation in most rodents. Seventy-seven miRNAs (Additional file 7, **File S5**) contain seeds without known homologues in vertebrates, of which twenty-four are derived from the opposite strand of known miRNAs. These may represent rat-specific miRNAs, although it cannot be excluded that these remain to be detected in other species.

### Tissue-specific miRNA profiles

**Figure 1:**
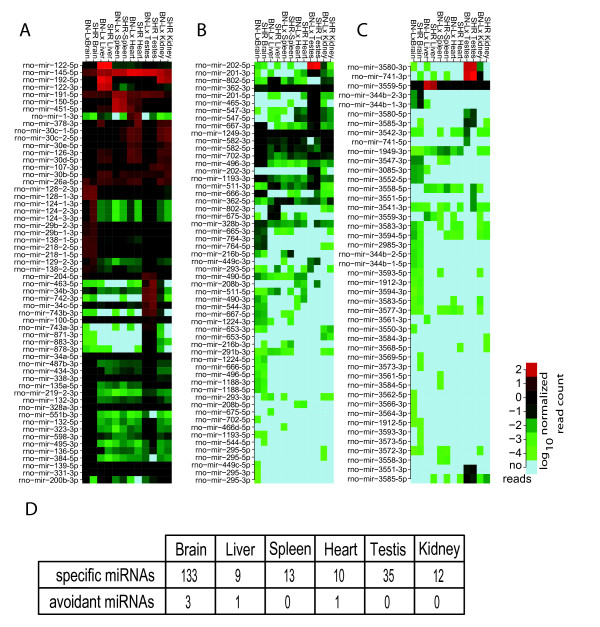
**miRNA tissue expression profiles**. **A-C) **Heatmaps show the expression (normalized, log_10_) of miRNAs. Each arm is represented separately, as are miRNAs in the reverse orientation (if applicable). In **(A)**, the first 10% of the heatmap is shown. miRNAs derived from loci that are homologous to miRBase human or mouse miRNA loci are shown in **(B)**. Completely novel miRNAs are shown in **(C)**. The legend is valid for the 3 heatmaps. **D) **Each tissue expresses a distinct set of miRNAs (tissue specific miRNAs). Also, some miRNAs are specifically not expressed in certain tissues (tissue avoidant miRNAs). miRNAs are specific in both replicates and the arbitrary, but strict, cutoff of 5-fold difference in expression was applied to determine specificity and avoidance.

miRNAs may act redundantly [42] or in concert [43,44]. In order to understand the function of miRNAs in specific tissues, it may therefore be helpful to identify global miRNA profiles. To be able to compare miRNA profiles, we normalized the miRNA reads per tissue and determined the relative expression of miRNAs among the six tissues. Comparisons between strains showed highly similar expression profiles, with the Spearman's ρ ranging from 0.88 (testis) to 0.95 (brain). The ρ among different tissue profiles was considerably lower, as expected [45], ranging from 0.62 (brain vs. Testis) to 0.87 (*e.g*. liver vs. spleen) (Additional file 8, **Figure S2**). We determined the number of reads that covered individual known miRNAs (Figure 1A, B, C and Additional file 9, **Figure S3**) and observed that 38% of the known miRNAs are ubiquitously expressed among all tissues that were assayed, whereas only 14% of the homologous and 2% of the novel miRNAs were identified in all examined tissues.

To systematically detect miRNAs that are differentially expressed between tissues, we applied a strict (5-fold) expression level difference that had to apply for each of the two strains. By applying this conservative cut-off we excluded subtle expression differences and expected to only include miRNAs that exert tissue specific regulatory roles. By demanding that this threshold is met in both strains, it is guaranteed that these differences are not specific for a specific genetic background. At this threshold, we identified a set of 212 tissue-specific miRNAs. The number of tissue-specific miRNAs varies considerably between tissues, ranging from 9 in liver to 133 in brain (Figure 1D and Additional file 10, **table S2**). Notably, we find 35 tissue-specific miRNAs in testis, the tissue from which we obtained least miRNA reads. This excludes coverage as a confounding factor in this analysis. By applying a 5-fold lower expression threshold in a specific tissue, we identified miRNAs that specifically lower expressed (or avoidant) in a given tissue. Five miRNAs are specifically depleted in brain, liver or heart (Figure 1D). These miRNAs are medium to highly expressed in all other tissues analyzed (Additional file 9, **Figure S3)**.

Some of these tissue-specific expression patterns are evolutionary conserved, for instance, the testis-specific *mir-202 *was previously reported to be testis-specific in chicken as well [46]. Other specifically expressed miRNAs that we verified are *mir-208*, which is known to be restricted to the heart [47] and *mir-122*, the most prominent miRNA in liver [48]. Brain, the most complex tissue in our dataset, also contains most tissue-specific and avoidant miRNAs. Eighteen miRNAs that have no known homologs are expressed in a tissue-specific manner, of which 5 are present in testis (the least covered datasets), 1 in liver, 1 in spleen and 11 in brain. The enrichment for both testis and brain-specific miRNAs in this dataset partially excludes lack of sequencing depth as a confounding factor. These results suggest that the emergence of expression of novel miRNAs occurs in a tissue-specific fashion. Furthermore, these novel tissue-specific miRNAs are found primarily in the brain, confirming earlier observations that miRNAs might play an important role in the evolutionary plasticity of the brain [49].

### Tissue-specific RNA editing

**Figure 2:**
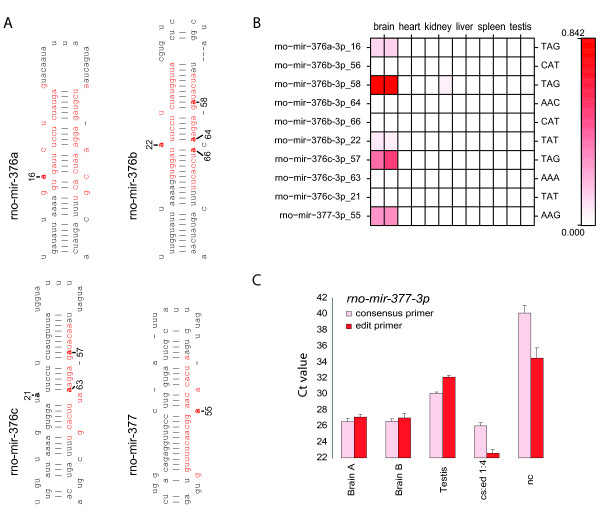
**Edited miRNAs**. **A) **miRNAs of which at least 10% was found to be edited. Shown are the precursors in their predicted folding structure. Red sequences represent miRNAs that are covered in our DGE profiles, bold "a"s along numbers indicate known and newly detected editing positions. **B) **Fractions of edited vs consensus miRNAs. Columns represent duplicates from each tissue, rows indicate miRNAs and edited position on the precursor. The red intensity reflects the fraction of edited miRNAs (edited miRNAs on this position/(edited + consensus miRNAs)). The triplets on the right side reflect the wild-type allele of which the middle adenosine is edited. **C) **qPCR data for *mir-377*. The first two bars describe results obtained from brain, the only tissue from which *mir-377 *was cloned. The ΔCt in brain corresponds to a ~2.7 fold higher expression of the consensus compared to the edited miRNA. Testis served as a negative input control. The specificity of the primers is indicated by the synthetic consensus and edited miRNA, mixed in a 1:4 ratio (cs: consensus, ed: edited). nc: primers only.

An auxiliary pathway to regulate miRNA expression and targeting is via the deamination of adenosines into inosines, a process known as RNA editing [50-52]. This process is mediated by adenosine deaminases acting on RNA (ADAR) [50]. During sequencing, inosines are read as guanines. To identify RNA editing we specifically identified A to G changes in otherwise perfectly mapped miRNA sequences and selected those miRNAs that are edited in at least 10% of the reads. After this selection we found that six miRNAs were subject to editing, including five positions in the miRNA seed region (Figure 2A). The most abundantly edited miRNA is *mir-376b*, in the brain (Figure 2B), fitting with the observation that in murine and human brains, *mir-376a/b/c *are targets of the RNA editing machinery [51,52]. We find the *mir-376a/c *isoforms to be edited as well, albeit to a lesser extent (Figure 2B). Importantly, in the case of *mir-376a-5p*, *mir-376b-5p*, *mir-376a-3p *and *mir-376-3p *the edited nucleoside resides in the seed region, which is instrumental in targeting the miRNA to its target and could therefore lead to an altered target spectrum.

In addition to known editing targets, such as *mir-376b*, we also identified the brain-specific *mir-377 *that was hitherto unknown to be edited. To quantify the degree of editing we verified editing of *mir-377 *by qRT-PCR analysis (Figure 2B, C, Additional file 11, **Table S3**). Based on dilution series we confirmed that the level of editing was ~37%, which is comparable to the level of editing as determined by DGE profiling.

### Strain specificity in the liver

**Figure 3:**
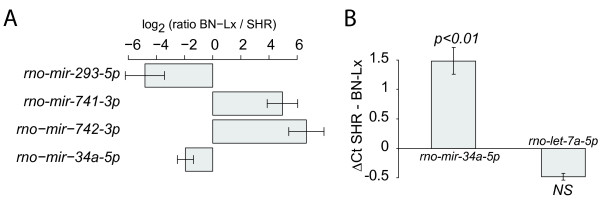
**Strain-specific miRNA expression**. **A) **Strain-differentially expressed miRNAs in the liver. The error bars indicate the estimated standard deviation (see methods for details). Only miRNAs of which the expression was significantly different (*p < 0.05*) are shown. The read frequency is normalized to the ocm (see methods for details). **B) **Differential expression of *mir-34a *confirmed by qPCR. The barplots show ΔCt values. This data was obtained from 3 × 3 replicates of each strain. The error bars show the combined standard deviation of all replicates for each miRNA. The student's T-test (2-sided) only assigned significance *(p < 0.01*) to mir-34a. NS, not significant.

The two strains from which we prepared small RNA libraries are the founder strains of the BXH/HXB recombinant inbred panel [39]. This is a well-established panel of rat strain that has been used for defining expression quantitative trait loci (eQTLs). As an initial indexation, we determined the liver miRNAs that are differentially expressed between the two strains. To estimate the biological variation between individuals, we performed DGE profiling on liver samples of three BN-Lx and three SHR rats. By estimating the variance in similarly expressed miRNAs within one strain (see methods for details) we found four miRNAs that were differentially expressed between strains *(p < 0.05) *in the liver, *i.e*. two increased in BN-Lx and two increased in SHR liver (Figure 3A). The two miRNAs that were more prominent in BN-Lx, *mir-742-3p *and *rno-mir-741-3p*, reside in the same genomic cluster and may thus originate from the same primary transcript. We found that the *mir-293-5p *homologue, which was not previously identified in the rat, was upregulated in SHR livers. Finally, we found that *mir-34a-5'*, which is known to be a transcriptional target of the pro-apoptotic p53 protein [53], was robustly increased in the SHR liver. We confirmed the differential expression of this miRNA by qRT-PCR (Figure 3B). The observed ΔCt of ~1.5 approximates the observed difference in our sequencing datasets (Figure 3A, B).

Expression of *rno-mir-741-3p *and *mir-742-3p*, which reside in the *mir-463 *genomic cluster and is thus likely to be expressed as a single transcript [54], is strain-specific. This suggests that miRNA-specific post-transcriptional regulation could strongly affect miRNA expression. In order to investigate the conservation of this cluster, we aligned its pre-miRNAs to the genome of the mouse. The mouse is the best annotated close relative of the rat and shares common ancestry with the rat 12-24 Mya [55]. We find that the *mir-463 *cluster is located in a syntentic region on chromosome X and is most prominently, but not uniquely, expressed in testis in both mouse and rat, indicating conservation of location and expression (Additional file 12, **Figure S4A, B) **[34]. *rno-mir-741 *is partially conserved; the syntenic mouse miRNA is *mmu-mir-741*. Notably the strongest diverged arm of *rno-mir-741*is the most abundant arm in our cloning data (Additional file 12, **Figure S4B**) and is the only miRNA arm that is described in *mmu-mir-741*, suggesting that expression is conserved while the sequence has diverged.

While most miRNA seeds on this cluster are strongly conserved between rat and mouse, divergence is an ongoing process that occasionally introduces novel functional miRNAs. Strain-specific processing of such miRNAs potentially precedes divergence: if by such regulation the miRNA expression is low, the miRNA itself can diverge without serious consequences. This may be one mechanism by which miRNAs evolve within this *mir-463 *cluster, in which the *rno-mir-741/mmu-mir-741 *ancestral miRNA has diverged between mouse and rat.

### piRNAs

**Figure 4:**
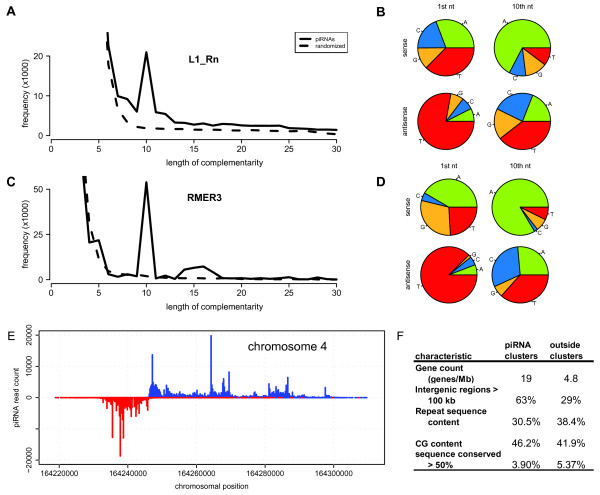
**piRNAs in the rat testis**. A, C) Complementarity of reads derived from repeats, calculated from the 5' terminus. A) L1 transposon, C) Retroviral repeat RMER3. Dashed lines indicate randomly selected short fragments from the same region. B, D) Nucleotide content of reads on position 1 and 10 that map to loci described in A and C. E) A typical pachytene piRNA cluster contains reads derived from bidirectional transcription originating from a centrally located common point of origin. Blue: sense, red: antisense. F) Characteristics of pachytene piRNA clusters.

The majority of small RNAs in the testis that we identified in our experiments were either repeat-derived or lacked annotation. Most likely these small RNA reads represent germline-specific piwi-assiociated RNAs (piRNAs). Because piRNAs function in genome maintenance by silencing of transposon sequences [16,17,56], we first investigated the piRNAs that mapped to annotated repeats like long tandem repeats an other transposon-derived repeats. Repeat-derived piRNAs are thought to be generated via a "ping-pong" mechanism [17]: a piRNA hybridizes to a target, which is subsequently cleaved along position 10 of the piRNA. By further processing, a novel piRNA is generated from the original target. In vertebrates, the maturation of piRNAs does not depend on *Dicer*, but on *piwi *homologues [18,56,57]. They archetypically start with a 5' uracil and posess an adenine on position 10, which suggest that they are *Argonaute*-dependent cleavage products [17]. As a result, one of the characteristics of the model is a 10 nt 5' region that is complementary between two piRNAs. We therefore tested for the presence of this characteristic signature, by selecting all piRNAs that mapped to a specific type of transposon (*i.e*. L1_Rn and RMER3) and looked for presence and length of overlap which, as expected, peaks at 10 nt (Figure 4A, C). Consistent with this overlap, we find the characteristic nucleotide distribution, with piRNAs mapping to the sense strand having a 5' terminal uracil, and piRNAs mapping to antisense strand having an adenine at 10^th ^position (Figure 4B, D). This signature is characteristic for piRNAs that have been generated via the ping-pong mechanism mentioned above.

An additional class of piRNAs is the pachytene piRNAs, which are found in large strand-specific clusters throughout the genome [20]. By employing a cluster identification algorithm based on the density of small RNA sequences within a specific region (see Methods), we found 365 strand specific clusters (Additional file 13, **Figure S5**, Additional file 14, **Table S4**). piRNA clusters are found on all the autosomes but are excluded from the X chromosome. The genomic distribution shows that clusters on the negative and the positive strand are often found in close proximity. Close inspection reveals that these clusters are often adjacent and hardly overlapping. After merging adjacent clusters we obtained 321 unique locations in the genome with piRNA clusters. Whenever we find two large non-overlapping piRNA clusters on opposite strands in close proximity these are found almost exclusively upstream of each other, with transcription pointing outwards (Figure 4E), suggesting a nucleation mechanism on the border between these two clusters [18].

The identified piRNA clusters allow for probing the characteristics of these sequences compared to the rest of the genome (Figure 4F). Only 35 RefSeq curated genes (n = 12,692) overlap with piRNA clusters, and only 4 reside completely within a cluster. The gene products cover a broad range of functions, but no common function could be pinpointed (data not shown). Interestingly, the gene density in these clusters is more than three-fold increased compared to regions outside of piRNA cluster. As previously reported, we confirm that rat piRNA clusters are strongly depleted in repeat sequences compared to the rest of the genome [18,19,21]. Although a high level of synteny among piRNA clusters has been reported [19], piRNA clusters diverge at an elevated rate [58]. Indeed, we find that the amount of conserved sequences, defined as having a phastCons score > 0.5 in a multiple alignment of 8 vertebrate species [59], showed a depletion of conserved sequences in these regions. In conclusion, piRNA clusters are genomic regions with distinct sequence characteristics. Their location and expression is maintained while their sequence diverges quickly. This would favor the idea that they target the region on which they are encoded [18]. Alternatively, it may well be that they generate small RNAs which act sequence independently.

## Conclusions

We performed small RNA DGE profiling for six rat tissues to expand the set of experimentally verified miRNAs and to gain biological insight into role of specific miRNAs. In total, we added 61 miRNA loci to the compendium of known rat miRNAs [40], providing experimental evidence for homologues of known mammalian miRNA as well as novel highly confident candidate miRNAs specific to the rat lineage. In addition to discovering novel miRNAs we quantified expression levels of miRNAs, creating a deep sequencing expression atlas of 6 tissues in two different rat inbred strains. This expression atlas can be an important instrument for understanding the evolution of miRNA regulation, *e.g*. conserved expression patterns among mammalian species, but also species-specific expression patterns. Our own experiments show strain-specific differences in miRNA expression and could form the basis for understanding this process at the molecular level.

Strain-specific miRNA expression patterns as determined by DGE can make important contributions to understand regulatory expression networks. The role of miRNAs in complex traits has not been fully addressed yet. Such traits are not inherited in a monogenic mendelian fashion, but are expected to be caused or modulated by subtle differences in multiple different types of regulation, including miRNA-mediated regulation. Our evidence for strain-specific miRNA expression could imply that eQTLs, which have been observed at the level of expression of protein-coding genes, could be partially explained by miRNAs, especially when linked regions span large genomic distances. In such cases, these so-called *trans *eQTLs could potentially be explained by differential miRNA expression (caused by a *cis*-effect of a causal local variant) and the linked region(s) could thus be expected to be enriched for miRNA target genes for this specific miRNA. In conclusion, strain-specific expression of miRNAs may help to identify and study these regulatory networks and facilitate target discovery for specific diseases.

In addition to transcriptional regulation, we confirmed the editing of known ADAR targets in rat, and discovered that, in addition to the known edited miRNAs, *mir-377 *is edited in rat brains. Since the physiology and neurobiology of the rat has been intensively studied, rat models could be an important experimental system for addressing the biological relevance of miRNA editing. Finally we provide an exhaustive list of piRNA candidates.

The small RNA inventories and profiles described here further improve the annotation of the rat genome, thereby facilitating functional genomics research and the investigation of the involvement of miRNAs in complex genetic traits.

## Methods

### Animal care and treatment

All animal experiments have been carried out according to the Dutch legal ethical guidelines and were approved by the Animal Care Committee of the Royal Dutch Academy of Sciences.

### MiRNA isolation and library preparation

Fresh tissues (i.e. brain, liver, spleen, heart, testis, kidney) from two rats (BN-Lx and SHR) and four additional liver samples (from BN-Lx and SHR) were directly frozen in liquid nitrogen and stored at -80°C until RNA isolation. Whole tissue was grained in liquid nitrogen and RNA isolation was performed by the Ambion Mirvana kit, according to the manufacturer's guidelines for total RNA isolation. After quality assessment on a 1% Agarose-TAE gel (data not shown), we prepared miRNA libraries by Ambion SREK kit according to the manufacturer's guidelines with the following specifications or deviations. We started with 2.5 μg total RNA from each sample and applied half of the suggested reagents for each step until PCR. We size fractionated total RNA in a 15-40 nt fraction on a 15% PAA denaturing (7 M Urea, TBE buffered) gel to collect small RNAs only. RNAs were eluted in 0.3 M NaCl fro 3 hrs at RT, precipitated at -80°C for 30 min in 3V EtOH, 10% 5 M ammonium acetate and 0.5 ul glycoblue (Ambion), spun down at 20 K G for 25 min at 4°C, washed in ice-cold 70% EtOH, spun down for 10 at 4°C and dissolved in nuclease-free H_2_O. We ligated adapters from adapter mix A for 2 hrs at 16°C. For all but testis (from both rats) and liver (from the SHR rat) we applied 14 cycli of PCR. For testis samples we applied 16 cycles of PCR and for SHR liver we applied 17 cycli. Directly after PCR we excised the amplified libraries from a 3% agarose-TAE gel, without exposing the libraries to UV light. The libraries were purified by the GFX PCR DNA and Gel Band purification kit (GE life sciences), according to the manufacturer's guidelines. The DNA size and quantity was measured on a Lonza 2.2% flash gel (Lonza) and Quant-iT dsDNA high sensitivity kit (Invitrogen), respectively (data not shown). Samples were mixed in to a 100 pg/μl solution. The emulsion PCR and Solid (AB) sequencing was performed as previously described [41]; the read length was set to 30 nt.

### Source specifications

The rat genome version RGSC3.4 and the mousse genome NCBI build 37 were used for mapping. miRNA identities and sequences were obtained from miRBase V12 [40,60]. All statistics and visualization was performed with the R package [61] or Microsoft excel. Genome sequences were obtained from Ensemble release 55 [62] and alignments performed by blast [63]. We used RNAshapes [64] to predict secondary structures of RNA. Scripts were written in perl [65] and R.

### Classifying small RNAs

All reads were submitted to the miRIntess small RNA analysis pipeline [38,49,66] (InteRNA Genomics B.V., Bilthoven, The Netherlands). The output of this analysis has been deposited on http://www.internagenomics.com/public/rat0812. This pipeline provided us with known miRNA reads and predicted miRNA reads with different levels of confidence. For instance, reads mapping to known miRNAs, but derived from the other arm, or from the opposite strand, were annotated with high confidence if they represented expected *Dicer *products and the pre-miRNA would form a stable hairpin. Homologous miRNAs were accordingly annotated. Similar rules applied to reads derived from yet unknown miRNAs, but the number of total reads should match at least 50 before these were assigned to be highly confident. Only low-confident novel miRNAs were excluded from the analysis, since predictions that fall into this category were indistinguishable from predictions performed on hairpins that had been randomly selected from genomic sequence (data not shown). Simultaneously the reads with a perfectly matching linker sequence were collected. The pre-miRNAs annotated by the mirIntess pipeline, except the category of low confident novel ones, were used to re-map the linker-trimmed reads, to obtain only perfectly mapping miRNA reads. Perl scripts are available upon request.

For the fine mapping of the small RNA reads we used the pre-miRNA hairpins that were identified by the miRIntess pipeline. Trimming of the sequencing linker was performed on the sequencing reads. Reads were subsequently remapped using SHRiMP [67], with the following settings (-h 50% -v 50% -s 1110111). Because this software package can natively map ABI colorspace reads, it has a higher sensitivity than the BLAST algorithm used in the miRIntess pipline. For all subsequent miRNA analyses, except the editing analysis (see below), only perfectly matching reads were used.

### Submission Gene expression omnibus, miRBase

Sequence reads have been submitted to GEO (accession number: GSE19054); microRNA sequences have been submitted to miRBase.

### Normalizing miRNA coverage

The coverage of each miRNA arm derived from a known miRNA locus (sense or antisense) was counted for each tissue. In almost all tissues we observed an extremely high coverage of a selected number of miRNAs. For instance, reads from the *rno-mir-122-5p *contribute to more than 20% of all reads from known miRNAs from liver. Such outliers would have a substantial impact on the outcome of differential expression when the reads would be normalized to the mean. To reduce the impact of outliers to the correction parameter, we removed the three highest covered miRNAs from the calculation of the outlier-corrected mean (ocm). The read coverage of each miRNA (known and novel) was divided by this ocm. We chose not to take the median as the correction parameter because of the high number of lowly covered miRNAs in our dataset.

### Editing analysis

To pick up edited miRNAs we scanned the mapped reads for A to G mutations. Because in ABI colorspace this requires two coherent colorbase changes, providing additional confidence that editing has indeed occurred in this miRNA.

### miRNA profiles

For profiles, reads were normalized according to the ocm (see earlier in text). We calculated the Euclidean distance between each miRNA and built a tree by hierarchical clustering. The log_10 _of each read frequency was visualized in heatmaps, for known, novel but homologous to known, and novel miRNAs.

For the determination of tissue-specific miRNAs, reads were normalized as described and the normalized frequency in one tissue should be at least 5-fold higher than in any other tissue, for each strain individually, to be included in our set of tissue-specific miRNAs. Similarly, this frequency should be 5-fold lower in one tissue than in any other tissue, for each strain individually, for the miRNA to be included as a tissue-avoidant miRNA.

### Differential miRNA expression in the liver

Read counts were normalized according to the ocm as described. We assigned significance α = 0.05, because of the low number of replicates and the high expected variation. We took means of all replicates from each strain: mean(BN-Lx) = *mB*, mean (SHR) = *mS*. We calculated average read frequency A = (mB + mS)/2 for both strains and relative expression values *D *= log_2_(mB/mS). Subsequently, the data was ordered based on A and using a sliding window approach, the mean and standard deviation were calculated for windows of 41 miRNAs surrounding every miRNA (with 20 identical values at both extremes). Those means and standard deviations were estimations, which we applied to calculate the *Z*-score of each *D*. We obtained the *P*-value defined by the one-sided normal distribution (*P*(X>*Z*)) and corrected *P*-values by the false discovery rate [68].

### qPCR analysis

The same RNA samples as those described in Methods section *MiRNA isolation and library preparation *were used. For the strain-specific miRNA expression, i.e. *mir-34a *and *let-7a*, we applied the miRNA taqman assay according to the manufacturer's guidelines, with 5 ng total RNA as input material. For *mir-377 *we designed circular probes of which the termini were complementary to the miRNA, leaving a gap on the edited site (RT-mir377: TTGTGTGATTCAaaacaagagaagaagtagatgtactagtgctgccacaacattaatcaagaAAAAGTTGCC) We applied 11 μg total RNA (biological sample) or 1 pmol (synthetic miRNAs). RT was performed (total volume: 20 μl; 2 μl buffer, 6 μmol dNTPs each, 1 pmol RT primer, incubation at 72°C for 5 min, add 100 U RT enzyme (promega, MMLV-), 37°C 40 min). Samples were diluted 10 × in H_2_O). PCR primers were diluted to 250 nM each (FWD-mir377-WT: CCCATGTTGAATCACACAAA, FWD-mir377-ED: CCCATGTTGAATCACACAAG, REV-mir377: TTGTGTGATTCAaaacaagag,). Quantitative PCR was performed in 12 μl sybrgreen (Biorad IQ), 10 μl primer mix and 3 μl template. On a thermocylcer (Biorad MyIQ), we performed qPCR (1× 96°C for 02:00; 2× 96.0°C for 00:15, 55.0°C for 00:15, 72.0°C for 00:30; 60× 96.0°C for 00:15, 60.0°C for 00:15, 72.0°C for 00:30, 80.0°C for 00:05; 1× 80.0°C for 10:00).

### mir-463 cluster comparison with mouse

Pre-miRNAs were aligned with the mouse genome by blastn (parameter deviating from default:-q -2). Loci with a minimal match length of 40 nt were investigated for a hairpin with approximately the same length of the original rat pre-miRNA. Only putative hairpins with at least 8 nt overlap with the cloned miRNA were included. Cloned miRNAs that aligned to the detected homologous locus had to match to at least one stem arm of the predicted pre-miRNA.

### Definition of piRNAs and piRNA clusters

We mapped reads to annotated repeats (LTRs and LINE elements) and counted the amount of possible reverse complementary interactions, for lengths from 1 to 30 nt. We also performed this analysis for a randomized set of 30-mers derived from the same repeat sequence.

After filtering out reads that are aligned to miRNAs, sn(o)RNAs, small cytoplasmic RNAs, rRNA genes or known coding genes, we found 271,382 unique start positions on the positive strand and 309,051 unique start positions on the negative strand. We have observed that distribution of these reads is non-random in the genome, *i.e*. clustered in certain locations. To rule out effects of clonality or cloning bias we only considered unique start positions. Regions that contain within a 2 kb window more than 20 unique start positions on the same strand are selected as the core of a piRNA cluster. From these piRNA core regions the start and end of the cluster is extended up- and downstream when a read is found on the same strand within 1 kb. This is continued until a read is found more than 1 kb from either start or end of the cluster.

## Authors' contributions

SL prepared the small RNA libraries, performed data analysis and wrote the manuscript. EB coordinated sequencing and handled the sequencer. EW performed data analysis and assisted in writing the manuscript. EC designed and coordinated the study. All authors read and approved the final manuscript.

## Supplementary Material

Additional file 1 **Table S1**. Read statistics. Click here for file

Additional file 2 **Figure S1**. Small RNA read description. Click here for file

Additional file 3 **File S1**. fastafile of all identified miRNAs in 6 tissues from the SHR and BN-Lx rat. Click here for file

Additional file 4 **File S2**. GFF of all known pre-miRNAs. Click here for file

Additional file 5 **File S3**. GFF of all homologous pre-miRNAs. Click here for file

Additional file 6 **File S4**. GFF of all novel pre-miRNAs. Click here for file

Additional file 7 **File S5**. fastafile of miRNAs without known seeds in vertebrates. Click here for file

Additional file 8 **Figure S2**. correlation between 6 datasets. Click here for file

Additional file 9 **Figure S3**. miRNAs derived from all known loci in the rat. Click here for file

Additional file 10 **Table S2**. Optimization qPCR for *mir-377*. Click here for file

Additional file 11 **Table S3**. Strain specific and avoidant miRNAs in six rat tissues. Click here for file

Additional file 12 **Figure S4**. evolving miRNAs in the rat *mir-463 *cluster. Click here for file

Additional file 13 **Figure S5**. pachytene piRNA clusters on the rat genome. Click here for file

Additional file 14 **Table S4**. piRNA clusters. Click here for file
