# Supplementary material for: Small RNA expression and strain specificity in the rat
Source: BMC Genomics. 2010 Apr 19;11:249. doi: 10.1186/1471-2164-11-249 (PMC2864251; doi:10.1186/1471-2164-11-249)
Supplement: Additional file 2 — Figure S1. Small RNA read description. [file 1471-2164-11-249-S2.PDF]

Figure S1 Linsen et al

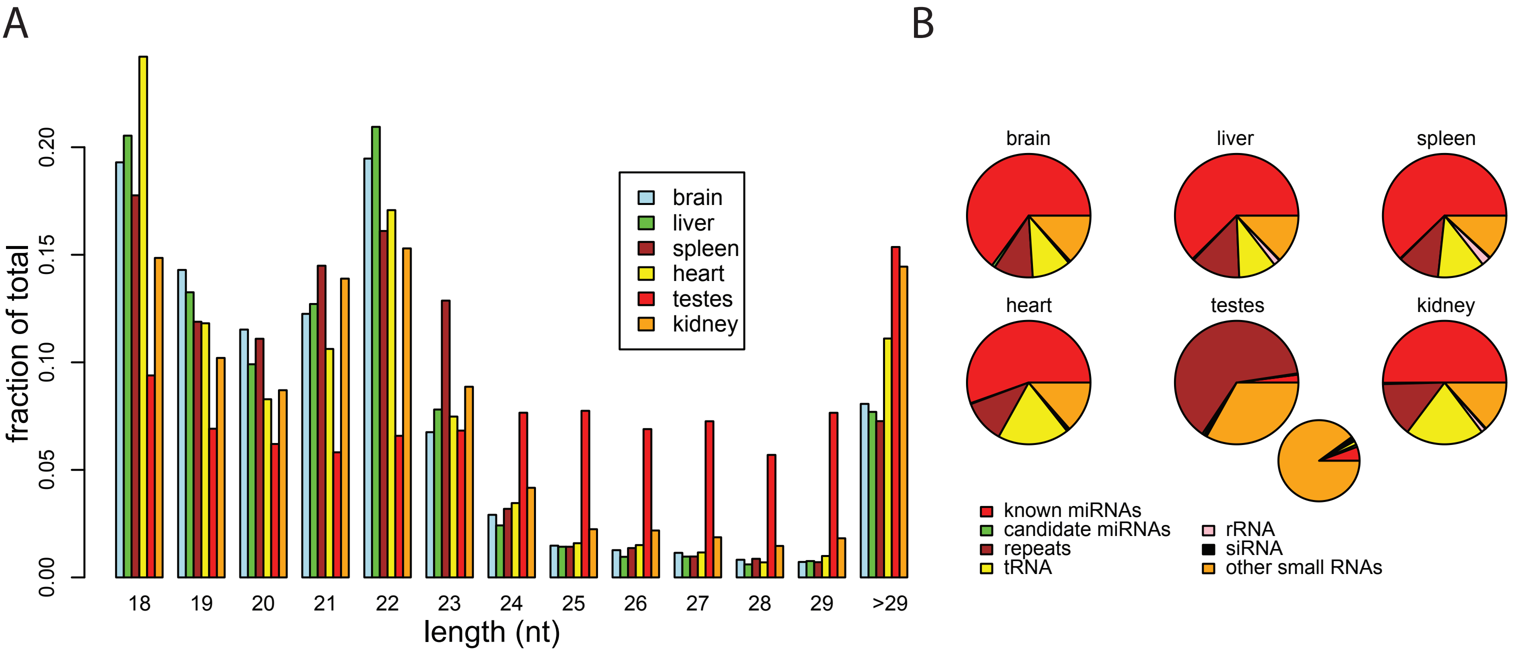

Figure S1. Small RNA read description. We sequenced small RNAs from six tissues from two rat strains. A) Length distribution of sequenced small RNAs. The fraction of the reads from tissues for each length is shown. The x-axis is limited from 18 to 30 nt , because only reads were included that mapped at least 18 nt to genomic sequence and the maximal read length was 30. B) Reads from the same tissues were combined (the inset under the testes chart shows the class distribution when repeats are removed) Known miRNAs: miRNAs from mirBase (v12); candidates: predicted miRNAs, homologs of known miRNAs, opposite-strand miRNAs, non-hairpin miRNAs i.e. mirtrons; other: sn(o)RNAs, scRNAs, sense RNAs, other hairpins, non-hairpin RNA, unclassified.
