# Supplementary material for: Small RNA expression and strain specificity in the rat
Source: BMC Genomics. 2010 Apr 19;11:249. doi: 10.1186/1471-2164-11-249 (PMC2864251; doi:10.1186/1471-2164-11-249)
Supplement: Additional file 8 — Figure S2. correlation between 6 datasets. [file 1471-2164-11-249-S8.PDF]

Figure S2 Linsen et al

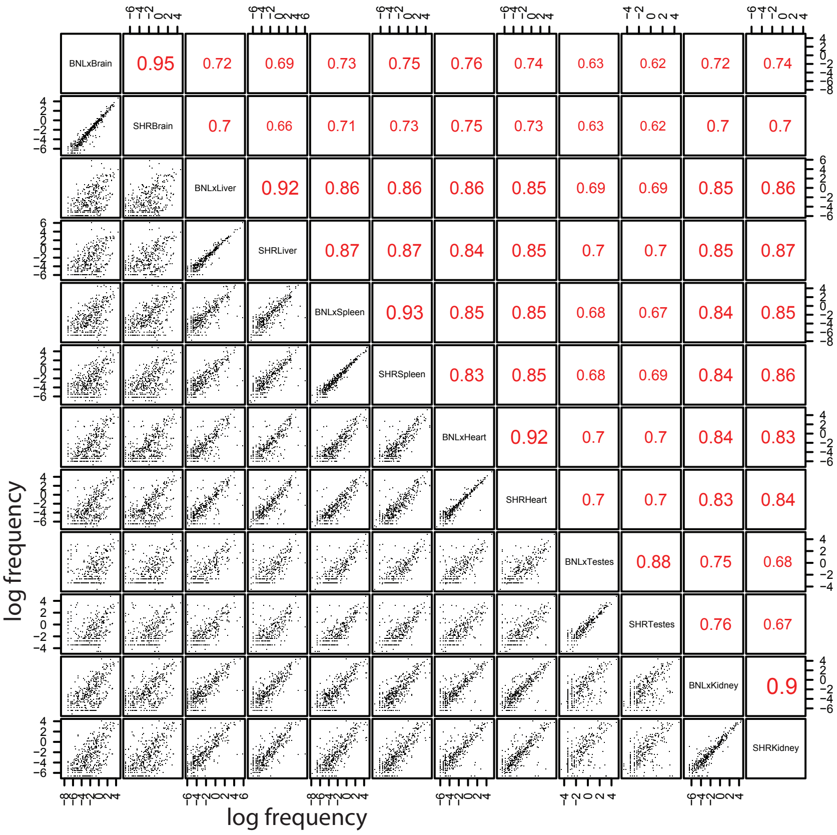

Figure S2. Correlation between 6 datasets. Each dataset is defined on the diagonal. Scatter plots show below the diagonal that the profiles are more reproducible among identical tissues than between different tissues. The correlation is quantified by the correlation coefficient (spearman's ρ) above the diagonal.
