# Supplementary material for: Small RNA expression and strain specificity in the rat
Source: BMC Genomics. 2010 Apr 19;11:249. doi: 10.1186/1471-2164-11-249 (PMC2864251; doi:10.1186/1471-2164-11-249)
Supplement: Additional file 9 — Figure S3. miRNAs derived from all known loci in the rat. [file 1471-2164-11-249-S9.PDF]

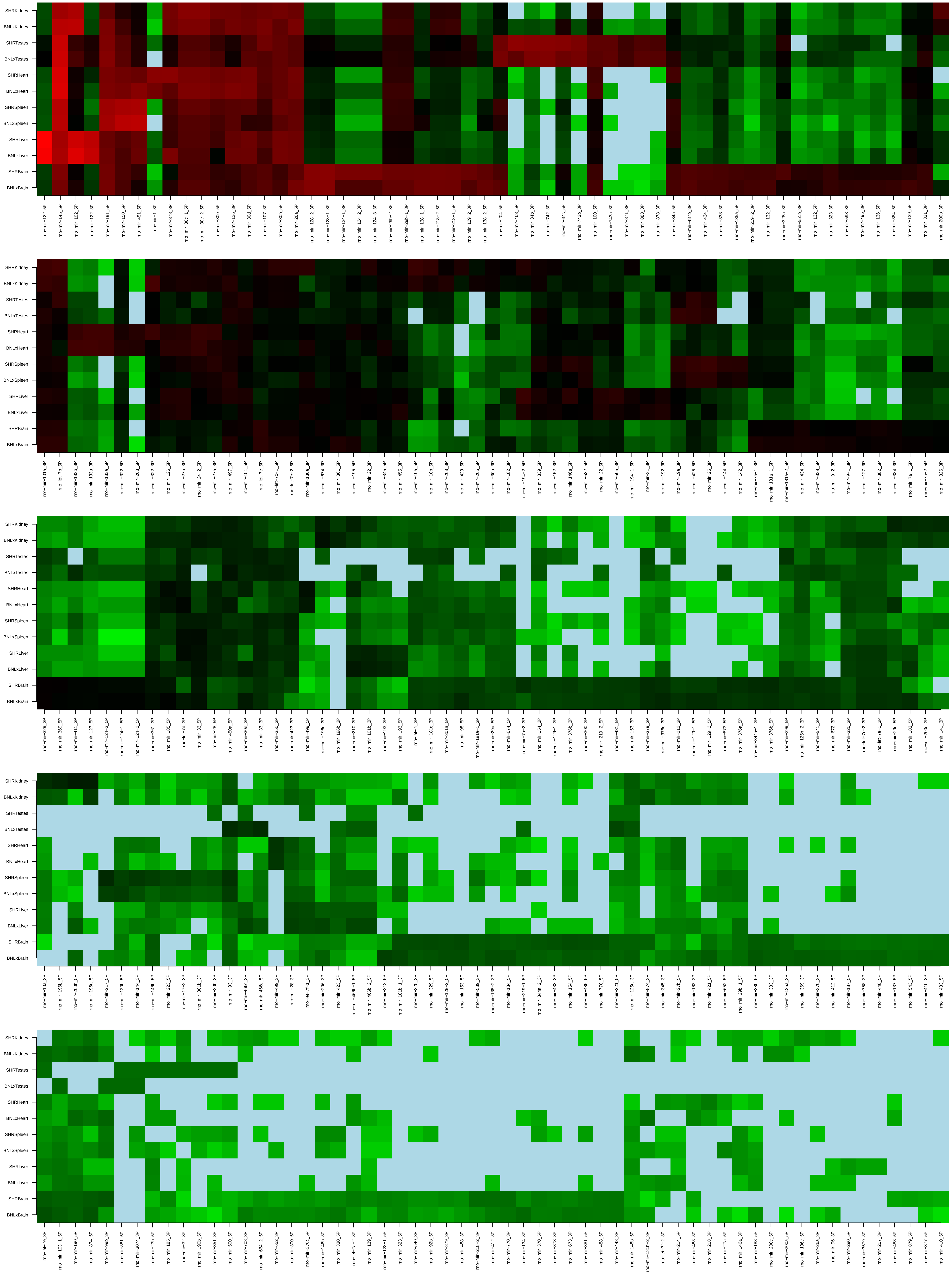
SHRKidney
BNLxKidney
SHRTestes
BNLxTestes
SHRHeart
BNLxHeart
SHRSpleen
BNLxSpleen
SHRLiver
BNLxLiver
SHRBrain
BNLxBrain

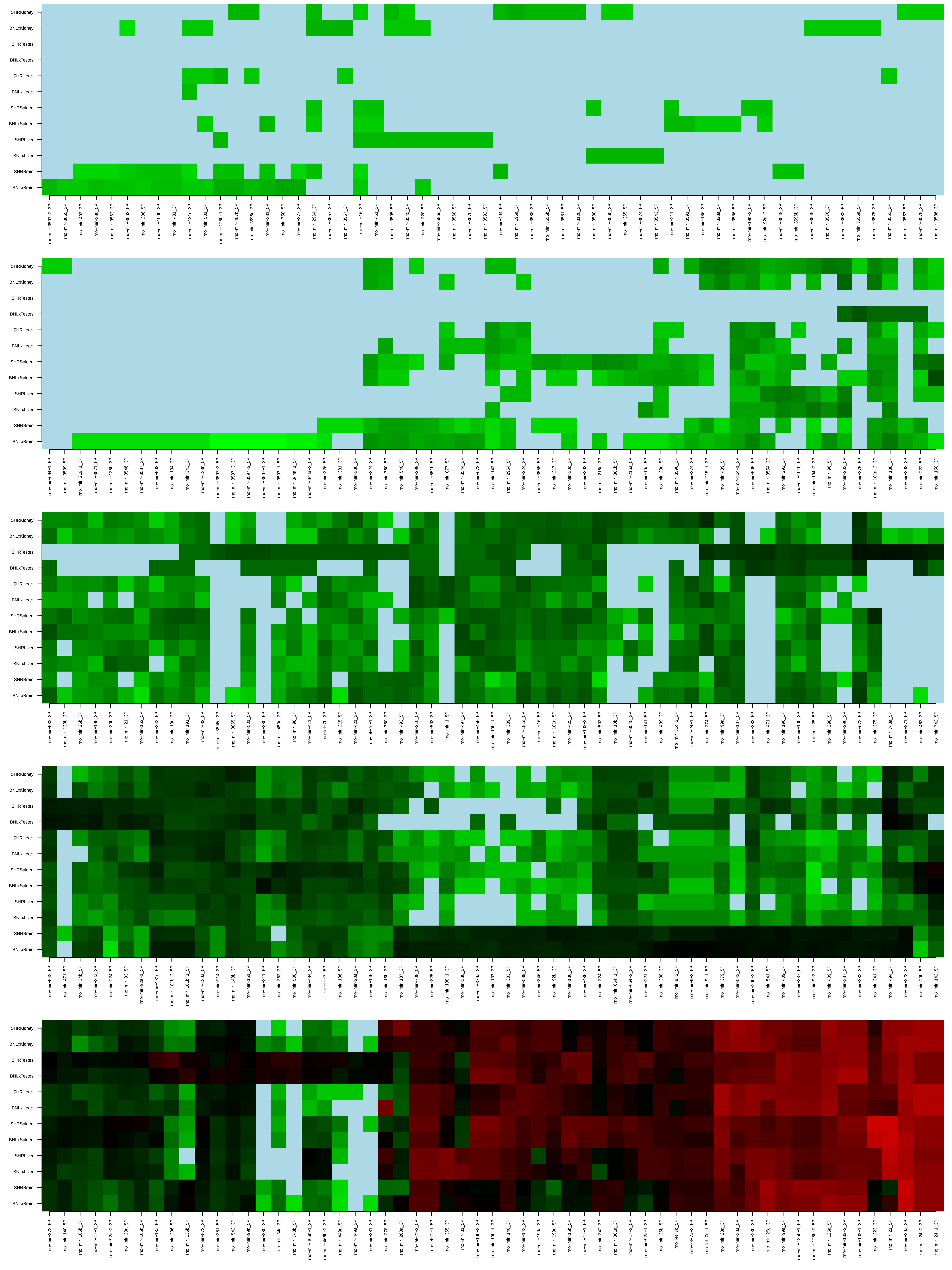
SHRKidney
BNLxKidney
SHRTestes
BNLxTestes
SHRHeart
BNLxHeart
SHRSpleen
BNLxSpleen
SHRLiver
BNLxLiver
SHRBrain
BNLxBrain

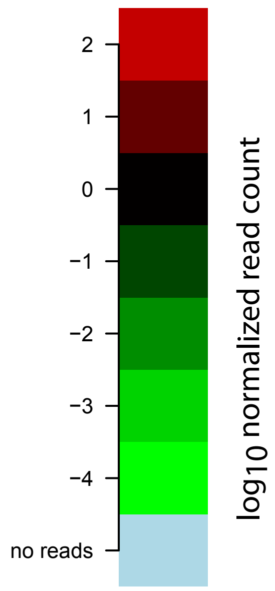

Figure S3 Linsen et al

Figure S3. miRNAs derived from all known loci in the rat. Heatmaps show, per arm, the normalized read coverage of each known miRNA locus.
