# Supplementary material for: Small RNA expression and strain specificity in the rat
Source: BMC Genomics. 2010 Apr 19;11:249. doi: 10.1186/1471-2164-11-249 (PMC2864251; doi:10.1186/1471-2164-11-249)
Supplement: Additional file 12 — Figure S4. evolving miRNAs in the rat mir-463 cluster. [file 1471-2164-11-249-S12.PDF]

Figure S4 Linsen et al

A

```
rat:   rno-mir-3551    X:152841101:152841182:-1
mouse: rno-mir-3551    X:64046475:64046557:-1
seed conserved: -
rat hp      (((((((..((((...((((((((((((..(((..((((......))))))..)))))))))))))...))))..)))))))
ratpre   5' TATGGGCAGTACTGTCTTCACCATGGTGTTTTTGACATAAGTAGCTATGAAATTCACCATGGTGGGTAAAGTAAGGCTCATA
ratclo                    TCTTCACCATGGTGTTTTTGACAT          TGAAATTCACCATGGTGGGTAAA
muspre      TATGGGCTGTATTTTATTCACCTTGGTGTTTATCAAGTGAATGACTATGAAAACCACCATGGTGGTTAAAGTAAAGTTCATA
mus hp      ((((..((.(((((((..((((.(((((.((.((((((.....))).))).)).))))).)))).)))))))).))..))))

rat:    rno-mir-741    X:152842633:152842728:-1
mouse:  mmu-mir-741    X:64049965:64050061:-1
seed conserved: -
rat hp      ((((((..(((.((.(((((((((((((..(((((.((.((((...........)))).)).)))))..))))))))))))).)).))))))))).
ratpre   5' AATGTCTCATGTGCATTGGTCTACATAGATTGGTACCTATCATGTAAAATAAGTGTGAAAGATGCCACGCTATGTAGATCAACGCTCATGACATTC
ratclo                           TACATAGATTGGTACCTATCA                AAAGATGCCACGCTATGTAGAT
muspre          TCTCATGTGCATTGATCTACGTAGATTGGTACCTATCATGTAAATCATGTAAGCATGAGAGATGCCATTCTATGTAGATTAAGGCTCATAACATTC
mus hp          ....(((.((.(((((((((((((.(((((.((.((((((...........)))))).)).))))).)))))))))))))).)).))).......
```

B

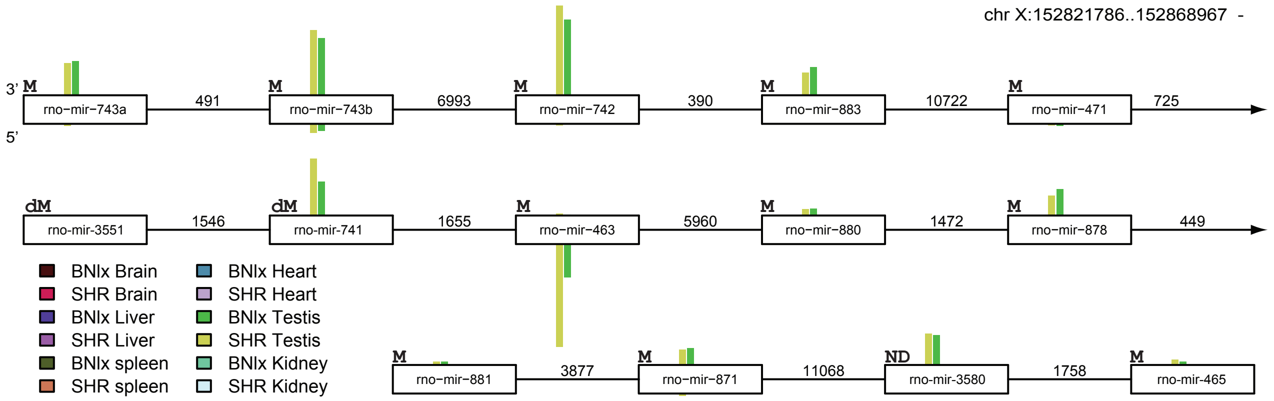

Figure S4. evolving miRNAs in the rat *mir-463* cluster. A) Divergence of miRNAs that are almost fully conserved in mouse. Some seed regions in these miRNAs have altered while the largest part of the miRNA sequences have been conserved. Grey planes highlight conserved sequence. hp: hairpin structure (matching brackets () indicate matching ribonucleotides in a hairpin, a dot is an unmatched ribonucleotide). pre: precursor DNA sequence. clo: cloned miRNA sequence from rat. B) The miRNAs from A reside within this testis-specifically expressed miRNA cluster. This cluster is highly conserved in mouse: all miRNAs that are emphasized with 'M' have been observed in mouse, in the identical order. ND: not detected. dM: miRNAs from A, diverged seed sequence in mouse. Coordinates and orientation are in the top right corner. The numbers between the blocks indicate the distance (bp) between each miRNA locus. The barplots above and under each block represent the miRNA-3' and 5' arm read frequency, respectively. The height reflects the normalized linear read frequency in arbitrary units. All tissues are shown, these miRNAs are substantially over-represented in testis.
