# Supplementary material for: Small RNA expression and strain specificity in the rat
Source: BMC Genomics. 2010 Apr 19;11:249. doi: 10.1186/1471-2164-11-249 (PMC2864251; doi:10.1186/1471-2164-11-249)
Supplement: Additional file 13 — Figure S5. pachytene piRNA clusters on the rat genome. [file 1471-2164-11-249-S13.PDF]

Fig S5 Linsen et al

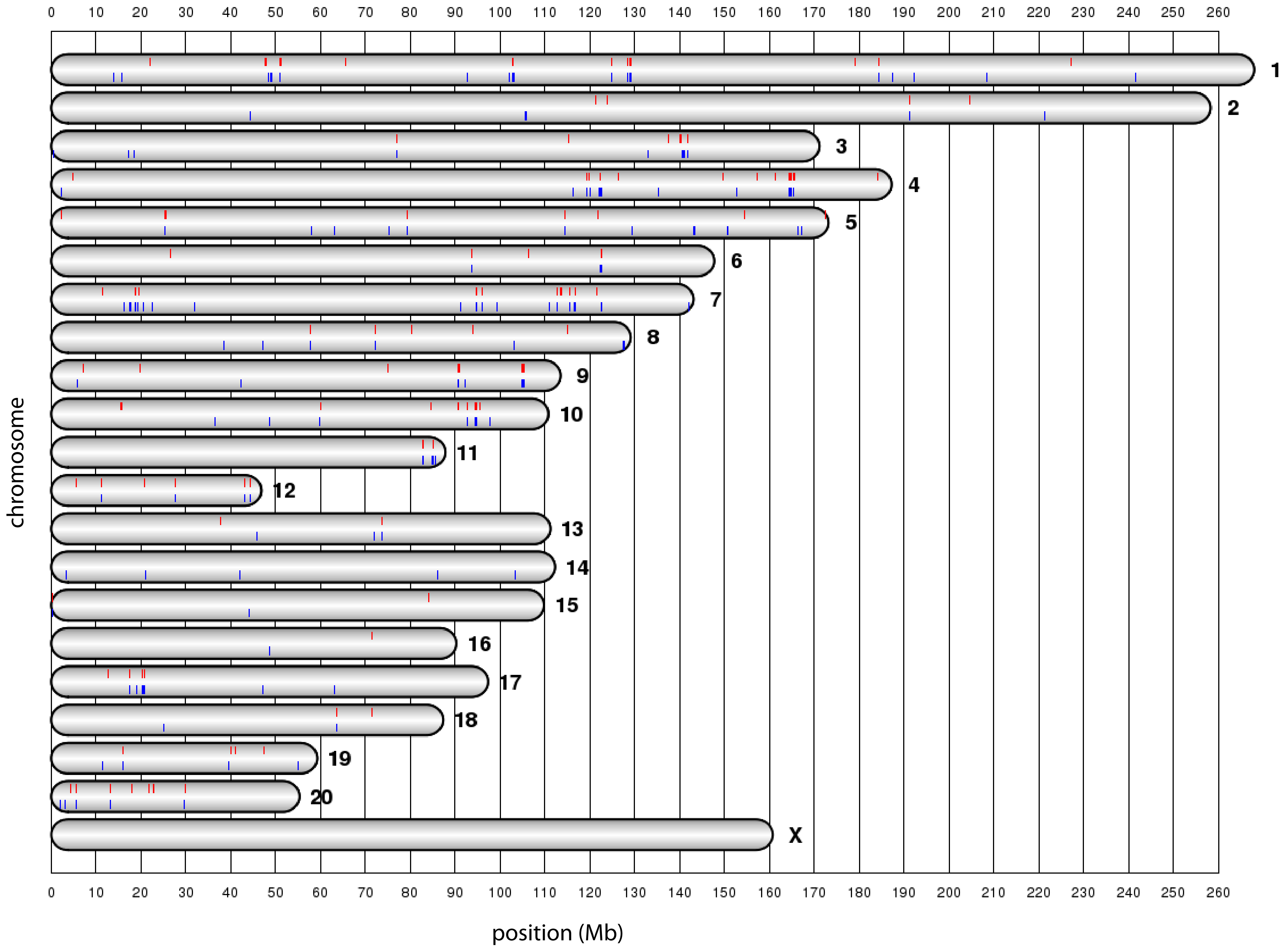

Fig S5. pachytene piRNA clusters on the rat genome.
Chromosome 1-20 and X are shown. Clusters from the "+" strand are depected in red, from the "-" strand depicted in blue.
